# The Complement of Projection Neurons Activated Determines the Type of Feeding Motor Program in *Aplysia*

**DOI:** 10.3389/fncir.2021.685222

**Published:** 2021-06-10

**Authors:** Colin G. Evans, Michael A. Barry, Jian Jing, Matthew H. Perkins, Klaudiusz R. Weiss, Elizabeth C. Cropper

**Affiliations:** ^1^Department of Neuroscience and Friedman Brain Institute, Icahn School of Medicine at Mount Sinai, New York, NY, United States; ^2^State Key Laboratory of Pharmaceutical Biotechnology, Institute for Brain Sciences, Collaborative Innovation Center of Chemistry for Life Sciences, Jiangsu Engineering Research Center for MicroRNA Biology and Biotechnology, Advanced Institute for Life Sciences, School of Life Sciences, Nanjing University, Nanjing, China

**Keywords:** mollusc, feeding, *Aplysia*, central pattern generators, command neuron

## Abstract

Multiple projection neurons are often activated to initiate behavior. A question that then arises is, what is the unique functional role of each neuron activated? We address this issue in the feeding system of *Aplysia*. Previous experiments identified a projection neuron [cerebral buccal interneuron 2 (CBI-2)] that can trigger ingestive motor programs but only after it is repeatedly stimulated, i.e., initial programs are poorly defined. As CBI-2 stimulation continues, programs become progressively more ingestive (repetition priming occurs). This priming results, at least in part, from persistent actions of peptide cotransmitters released from CBI-2. We now show that in some preparations repetition priming does not occur. There is no clear seasonal effect; priming and non-priming preparations are encountered throughout the year. CBI-2 is electrically coupled to a second projection neuron, cerebral buccal interneuron 3 (CBI-3). In preparations in which priming does not occur, we show that ingestive activity is generated when CBI-2 and CBI-3 are coactivated. Programs are immediately ingestive, i.e., priming is not necessary, and a persistent state is not induced. Our data suggest that dynamic changes in the configuration of activity can vary and be determined by the complement of projection neurons that trigger activity.

## Introduction

Many central pattern generators (CPGs) receive input from multiple projection neurons. In some cases, these neurons are each clearly associated with the induction of a distinct behavior, e.g., one neuron (or set of neurons) triggers forward locomotion, whereas a second neuron (or set of neurons) triggers backward locomotion (Takagi et al., 2017; Carreira-Rosario et al., 2018; Bidaye et al., 2020). In many other situations, this is not the case. Multiple projection neurons are associated with the induction of a single type of motor activity (e.g., Kristan and Shaw, 1997; Kemenes et al., 2001; Beenhakker and Nusbaum, 2004; Hedrich et al., 2009). A question that then arises is, what is the unique functional role of each projection neuron activated?

We address this issue in experiments conducted in the feeding network of the mollusc *Aplysia californica*. This network generates both ingestive and egestive motor programs (Cropper et al., 2017). When activity is ingestive the food grasping organ, the radula, closes as it retracts into the buccal cavity. This combination of radula movements pulls food in (Morton and Chiel, 1993a, b). When activity is egestive, the radula closes as it protracts, which pushes food out. Radula movements result from the activation of a feeding central pattern generator (CPG) that is located in the buccal ganglion. Projection neurons that provide input to the feeding CPG are cerebral buccal interneurons (CBIs) that have somata in the cerebral ganglion (Rosen et al., 1991).

The most well-characterized CBI is CBI-2 (Rosen et al., 1991; Zhang et al., 2020). CBI-2 is a cholinergic neuron that contains the neuropeptides feeding circuit activating peptide (FCAP; Koh et al., 2003) and cerebral peptide 2 (CP-2; Phares and Lloyd, 1996; Morgan et al., 2000). CBI-2 receives afferent input from neurons activated by food, and when it is stimulated it reliably triggers motor programs (Rosen et al., 1991). However, if preparations are rested prior to CBI-2 stimulation, the first cycle of activity generated most commonly has “intermediate” characteristics (e.g., Proekt et al., 2004; Friedman and Weiss, 2010). For example, radula closer motor neurons fire at a low frequency during both the radula protraction and retraction phases of the motor program, and functional closing does not occur.

Previous experiments identified one manipulation that can convert intermediate activity to ingestive. Namely, when CBI-2 is stimulated with short interburst intervals so that multiple cycles of activity are generated there can be progressive, gradual increases in the firing frequency of the radula closer motor neurons during retraction, i.e., ingestive repetition priming occurs (Proekt et al., 2004). When priming occurs, effects of repeated CBI-2 stimulation persist, and an ingestive state is created that lasts approximately 20–40 min (Friedman and Weiss, 2010; Cropper et al., 2014; Perkins et al., 2018, 2019). This persistence is presumably a consequence of the fact that peptides released by CBI-2 exert second messenger mediated effects (Friedman and Weiss, 2010; Perkins et al., 2018, 2019).

In this study, we demonstrate that there are some preparations in which CBI-2 induced repetition priming does not occur. Even with repeated stimulation of CBI-2, motor programs remain intermediate. In these preparations, we show that activity does, however, become ingestive if a second projection neuron (CBI-3) is coactivated with CBI-2. CBI-3 is a GABAergic neuron that does not contain the peptides present in CBI-2 (FCAP and CP-2; Jing and Weiss, 2001; Morgan et al., 2002; Jing et al., 2003). With CBI-3 coactivation, alterations in motor activity occur immediately and do not persist. Thus, our data suggest that the complement of projection neurons activated may determine how quickly network activity can be configured and reconfigured.

## Materials and Methods

### Animals

Adult sea slugs (*Aplysia californica*) were purchased from Marinus Scientific (Long Beach, CA) and housed in artificial seawater (ASW; Instant Ocean, Cincinnati, OH) at 15–18°C. *Aplysia* are hermaphrodites, so are both male and female. Animals weighing 150–200 g were anesthetized by injection of 60–120 ml isotonic (i.e., 337 mM) MgCl_2_. Cerebral and buccal ganglia were removed and desheathed in a solution containing 50% ASW (in mM: 460 NaCl, 10 KCl, 55 MgCl_2_, 11 CaCl_2_, and 10 HEPES buffer, pH 7.6) and 50% isotonic MgCl_2_. Desheathed ganglia were then superfused with ASW at 0.3 ml/min and maintained at ~14–17°C for about an hour prior to the start of experiments.

### Electrophysiological Recordings

Intracellular recordings were obtained using glass micropipettes filled with a 0.6 M K_2_SO_4_ and 60 mM KCl electrolyte solution. Electrodes were fabricated using a Flaming/Brown micropipette puller (Sutter Instrument Co., Novato, CA) to yield a final resistance of 6–10 MΩ. Electrodes were held in HS-2A headstages (Molecular Devices, San Jose, CA) connected to AxoClamp 2B amplifiers (Molecular Devices). Extracellular recordings were obtained from the I2 nerve (I2N) using polyethylene suction electrodes connected to a Model 1700 differential AC amplifier (bandpass 0.1–1 kHz; A-M Systems; San Diego, CA). Both intracellular and extracellular signals were digitized using a Digidata 1320A (Molecular Devices). Data were acquired using AxoScope software (Molecular Devices).

### Induction of Motor Programs

To trigger motor programs CBI-2 was intracellularly stimulated during the protraction phase of the motor program at 9 Hz with brief (10 ms) current pulses to elicit one-for-one action potentials. To induce ingestive repetition priming, we stimulated CBI-2 so that ~7 cycles of motor activity were triggered with 30 s between the termination of the retraction phase of one cycle and the initiation of the protraction phase of the following cycle (Friedman and Weiss, 2010). In preparations in which CBI-3 was activated, it was intracellularly stimulated during the protraction phase of the motor program at 15 Hz with brief (10 ms) current pulses to elicit one-for-one action potentials. In preparations in which we triggered more than one series of cycles, we waited at least 1 h in between trials. Previous work has demonstrated that this time period is sufficient to allow effects of repetition priming to dissipate (Perkins et al., 2018).

### Classification of Feeding Motor Programs

Motor activity was classified as has been previously described. The protraction phase of the motor program was monitored by recording from the I2 nerve, which contains the axons of protraction motor neurons (Hurwitz et al., 1996) Retraction was defined by the cessation of activity in the I2 nerve and by the end of depolarization of B8. Radula closing was monitored by recording from the B8 motor neurons (Morton and Chiel, 1993a, b). The activity was classified as ingestive when the B8 firing frequency during protraction was less than 3.5 Hz, the firing frequency during retraction was greater than 4.5 Hz, and the ratio of the two numbers was less than 0.65 (Morgan et al., 2002). The activity was classified as egestive when the B8 firing frequency during protraction was >3.5 Hz, the firing frequency in retraction was <2.5 Hz, and the ratio of the two numbers was greater than 2 (Morgan et al., 2002). All other motor programs were classified as intermediate.

### Statistics

Data were analyzed in Clampfit and organized in Excel. Data were plotted and analyzed in Prism (GraphPad Software, San Diego, CA). Error bars indicate SEMs and the significance level was set at *p* < 0.05 (*p < 0.05; **p < 0.01; ***p < 0.001; ****p < 0.0001; n.s., *p* > 0.05). Where applicable, measurements under different conditions were treated as repeated measures as indicated in the text. Throughout the results, *n* refers to the number of preparations.

## Results

**Figure 1:**
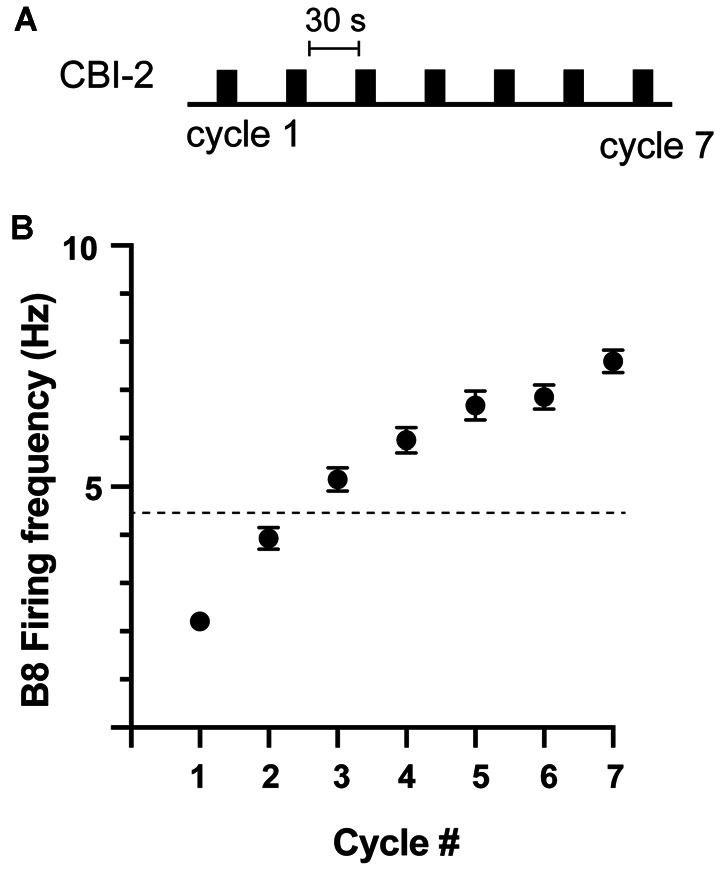
Ingestive repetition priming. **(A)** Protocol used to induce ingestive repetition priming. Cycles of activity were triggered by stimulating CBI-2 with an inter-burst interval of ~30 s. **(B)** The B8 firing frequency during radula retraction in preparations in which ingestive priming was observed. The activity was classified as described in the methods. The dashed line marks the B8 firing frequency at which functional radula closing occurs (Friedman et al., 2009). This is approximately where activity becomes ingestive. Plotted are means ± SEMs (individual values are shown in Figure 2A; *n* = 78).

**Figure 2:**
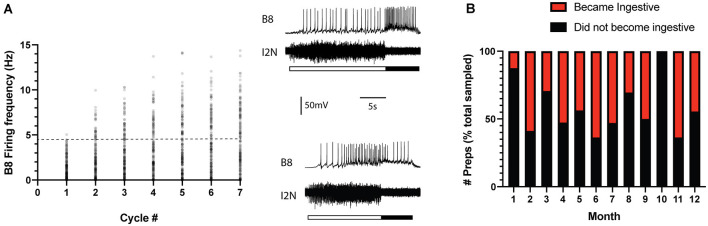
Ingestive priming occurred in some preparations but not in others. **(A)** Preparations were tested for ingestive priming using the protocol shown in Figure 1A. Scatter plot of the B8 firing frequency during the radula retraction phase of the motor program. The activity was classified as described in the methods. The dashed line marks the B8 firing frequency at which functional radula closing occurs (Friedman et al., 2009). This is approximately where activity becomes ingestive. Insets to the right are sample recordings. In both cases, the top traces are intracellular recordings from B8 and the bottom traces are extracellular recordings from the I2 nerve (I2N). The filled bar below the traces marks the radula retraction phase of the motor program. The open bar marks the radula protraction phase. Data were obtained from 186 preparations. The activity became ingestive in 78 preparations and remained intermediate in 108 preparations. **(B)** Data in panel A plotted to indicate whether ingestive priming did (red) or did not (black) occur during the different months of the year. Data are plotted as a % of the total number of preparations sampled per month. Note that there was no clear seasonal pattern.

Previous studies established that ingestive repetition priming can occur if CBI-2 is stimulated so that it triggers multiple cycles of motor activity with a relatively short interval in between periods of activity (Figure 1A; Proekt et al., 2004, 2007; Friedman et al., 2009; Friedman and Weiss, 2010; Dacks et al., 2012; Siniscalchi et al., 2016; Perkins et al., 2018). This is presumably a consequence of the fact that repetition priming is mediated by the actions of modulatory neurotransmitters released under these conditions (Cropper et al., 2014). We conducted experiments in which we stimulated CBI-2 using an established protocol and classified activity as others have (see “Materials and Methods” section). We found that repetition priming occurred in some of the preparations we tested (Figures 1B, 2A; one-way repeated measures ANOVA; *n* = 78; *F*_(6, 462)_ = 156.5, *p* < 0.0001). However, in other preparations, we found that activity did not become ingestive (Figure 2A; *n* = 108).

To determine whether the absence of priming was simply a reflection of a transient difference in the initial state, preparations in which repetition priming did not occur were retested (i.e., a second series of cycles was triggered by stimulating CBI-2). In 22/108 preparations, activity did subsequently become ingestive. However, in the majority (87/108) of preparations it did not.

*Aplysia* develop from eggs then go through different stages of development throughout their 1-year lifespan (e.g., Carefoot, 1987). They generally reproduce in the late spring and summer and shortly thereafter die. To determine whether the inability to prime was predominantly associated with the *Aplysia* mating season, we grouped data by date (i.e., the month of the year in which the experiment was conducted; Figure 2B). These data show that the absence of priming was not associated with a particular season, i.e., it was observed throughout the entire year ($X_{\left(1,N=108\right)}^{2}$ = 1.025, *p* = 0.3113).

The preparations tested in these experiments came from adult animals that had successfully matured in the wild. Clearly, they were capable of ingesting food. Previous studies demonstrated that there is a second projection neuron that is electrically coupled to CBI-2 that is also associated with the induction of ingestive activity (Morgan et al., 2002). Data indicate that this neuron, cerebral buccal interneuron 3 (CBI-3), is not a functional duplicate of CBI-2. For example, CBI-2 and CBI-3 have different morphological features (Rosen et al., 1991) and differ biochemically (Phares and Lloyd, 1996; Morgan et al., 2000, 2002; Jing and Weiss, 2001; Jing et al., 2003; Koh et al., 2003).

**Figure 3:**
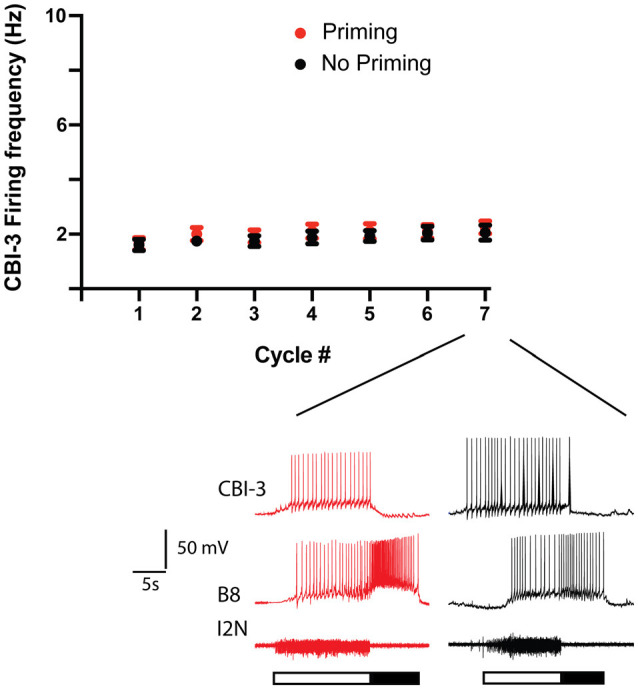
CB-3 recruitment in preparations in which priming was observed and preparations in which it was not. Seven cycles of activity were triggered by stimulating CBI-2 using the protocol shown in Figure 1A. The mean CBI-3 firing frequency + the SEM is plotted for preparations in which priming occurred (red, *n* = 15) and for preparations in which priming did not occur (black, *n* = 13). The top two traces in the sample recordings below the scatter plots are intracellular recordings, the bottom trace is an extracellular recording. The filled bar below the traces marks the radula retraction phase of the motor program, the open bar marks the radula protraction phase. Note that CBI-3 fired at a low frequency in both situations.

To determine whether differential recruitment of CBI-3 could account for differences in priming, we measured the CBI-3 firing frequency both in preparations in which ingestive priming was observed and in preparations in which it was not (Figure 3). CBI-3 fired at a relatively low frequency during all seven cycles of activity in both cases (i.e., it fired at ~2 Hz; two-way ANOVA; *n* = 15 for preparations in which priming occurred, *n* = 13 for preparations in which priming failed; *F*_(1, 182)_ = 1.628, *p* = 0.2035 for priming vs. non priming; *F*_(6, 182)_= 1.107, *p* = 0.3597 for cycle number; *F*_(6, 182)_= 0.0567, *p* = 0.9992 for the interactive effect). These data indicate that CBI-3 was not preferentially recruited in preparations in which priming occurred.

**Figure 4:**
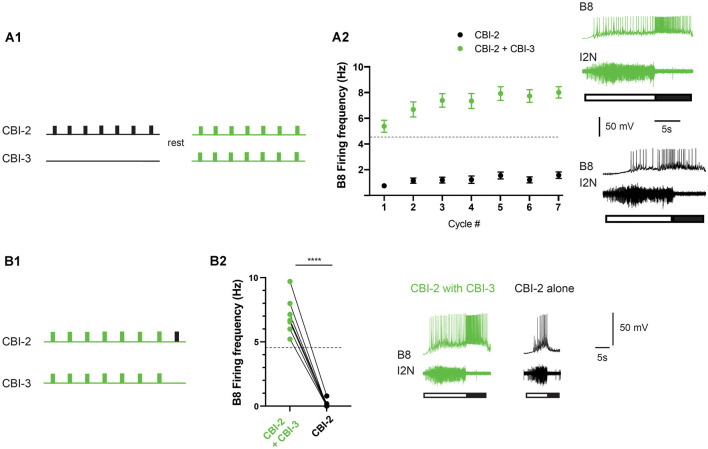
CBI-2 and CBI-3 coactivation. **(A)** CBI-3 coactivation makes activity immediately ingestive. Seven cycles of activity were triggered using CBI-2. If repetition priming was not observed, preparations were rested and then CBI-2 and CBI-3 were coactivated **(A1)**. Plotted are means + SEMs for the B8 firing frequency during radula retraction when CBI-2 was stimulated on its own (black) and when CBI-2 and CBI-3 were coactivated (green; A2; *n* = 26). Activity was classified as described in the methods. The dashed line marks the B8 firing frequency at which functional radula closing occurs (Friedman et al., 2009). This is approximately where activity becomes ingestive **(A2)**. The top traces in the sample recordings are intracellular recordings, the bottom trace is an extracellular recording. The filled bar below the traces marks the radula retraction phase of the motor program, the open bar marks the radula protraction phase. **(B)** Effects of CBI-3 coactivation are not persistent. Seven cycles were triggered by CBI-2/CBI-3 coactivation (green) and then an eighth cycle was induced by stimulating CBI-2 alone (black; *n* = 7) **(B1)**. In all preparations, there was an immediate decrease in the B8 firing frequency **(B2)**. The top traces in the sample recordings are intracellular recordings, the bottom traces are extracellular recordings. The filled bar below the traces marks the radula retraction phase of the motor program, the open bar marks the radula protraction phase. ****p < 0.0001.

CBI-3 is like CBI-2 in that it receives afferent input. When food contacts feeding structures in semi-intact preparations, the CBI-3 firing frequency is variable (it ranges between ~3 and 10 Hz; Wu et al., 2014). Thus, it is generally above 2 Hz. To determine whether stimulating CBI-3 at a higher frequency could make activity ingestive in preparations in which priming failed, we conducted experiments in which we attempted to induce priming by repeatedly stimulating CBI-2. If priming failed, preparations were rested and activity was triggered by co-activating CBI-2 and CBI-3 (Figure 4A). CBI-3 was stimulated at 15 Hz. This is approximately twice the mean frequency recorded in semi-intact preparations to compensate for the fact that only one of the two CBI-3 was stimulated (for technical reasons). Stimulation of CBI-3 had a significant effect (Figure 4A; two-way ANOVA; *n* = 26; *F*_(1, 50)_ = 140.3, *p* < 0.0001 for the effect of CBI-3 stimulation; *F*_(6, 300)_ = 16.18, *p* = < 0.0001 for cycle number; *F*_(6, 300)_ = 5.337, *p* = < 0.0001 for the interactive effect). Interestingly, with CBI-3 stimulation, activity was immediately ingestive, e.g., the mean frequency during cycle one was above the frequency that produces functional radula closing (4.5 Hz). This is distinctly different from the changes in radula closer activity that are observed during priming (Figure 1B). To determine whether this effect was simply due to effects of repeated stimulation of CBI-2, we reanalyzed a subset of the data obtained from experiments in which priming was not observed. In particular, we measured the B8 firing frequency during retraction in preparations in which these data were recorded for all seven cycles of activity when a second series of motor programs was generated (i.e., preparations were stimulated using the protocol shown in Figure 4A without CBI-3 co-activation). When we compared series 1 to series 2 data there was no significant difference (two-way ANOVA; *n* = 10; *F*_(1, 18)_ = 0.115, *p* = 0.7384 for series 1 vs. series 2; *F*_(3.633, 65.4)_ = 5.634, *p* = 0.0009 for cycle number; *F*_(6, 108)_ = 1.395, *p* = 0.2233 for the interactive effect).

When activity becomes ingestive as a result of repetition priming induced by CBI-2 stimulation, a relatively persistent state is created that lasts ~20–40 min. To determine whether effects of CBI-3 stimulation also persist we conducted experiments in non-priming preparations in which we triggered seven cycles of activity by co-activating CBI-2 and CBI-3 followed by one cycle in which activity was triggered by CBI-2 alone (Figure 4B). When CBI-3 coactivation ceased there was an immediate decrease in the B8 firing frequency and activity was no longer ingestive (Figure 4B; *n* = 7; paired *t*-test, *t*_(6)_ = 14.7, *p* < 0.0001). This indicates that CBI-3 coactivation does not induce a persistently ingestive state.

## Discussion

Behavior generating networks often receive input from multiple projection (or descending) neurons. These projection neurons can be multiple copies of one cell type (Colton et al., 2020). In other situations, they are functionally distinct neurons that are differentially activated by afferent input and in turn, each triggers a functionally distinct motor output. For example, P9 neurons in *Drosophila* trigger forward walking with ipsilateral turning and are necessary for a male to pursue a female during courtship. On the other hand, BPN drives straight, forward walking and is not required for courtship (Bidaye et al., 2020).

In other situations, projection neurons are activated during more than one behavior. For example, in lamprey, a number of reticulospinal neurons activated during swimming are also activated during crawling (Zelenin, 2005). Many of the swimming command neurons in the leech are excited during shortening (Kristan and Shaw, 1997). In some cases, coactivation of projection neurons appears to reflect a modular design (e.g., Huang et al., 2013). In this situation one group of projection neurons is responsible for one component of a behavior, whereas a second group is responsible for a second component. Projection neurons are therefore coactivated since coactivation is necessary to generate a complete behavior. The situation we describe differs in that CBI-2 and CB-3 do not recruit different modules; both modify B8 activity to make motor programs ingestive.

CBI-2 is a cholinergic neuron that contains two neuropeptides, FCAP and CP-2 (Phares and Lloyd, 1996; Morgan et al., 2000; Koh et al., 2003). Previous studies demonstrated that the FCAP/CP-2 released from CBI-2 plays an important role in reconfiguring the motor circuit. At loci that have been examined, FCAP/CP-2 potentiates synaptic transmission or increases excitability, and effects are second messenger (i.e., cAMP) mediated (Koh et al., 2003; Koh and Weiss, 2005, 2007; Friedman and Weiss, 2010; Dacks and Weiss, 2013; Siniscalchi et al., 2016; Perkins et al., 2018, 2019; Zhang et al., 2018). Effects persist for minutes, which presumably accounts for the slow dynamics that are observed during ingestive repetition priming. In contrast, CBI-3 is a GABAergic neuron that does not contain FCAP or CP-2 but does contain a third peptide, APGWamide (Morgan et al., 2000; Jing and Weiss, 2001; Jing et al., 2003).

A previous study demonstrated that CBI-3 modifies motor programs by suppressing the activity of egestive interneurons (Jing and Weiss, 2001). One of these interneurons (B20) fires during the radula protraction phase of the motor program. This is when CBI-3 itself is active. Effects of CBI-3 on B20 are due to GABA release (Jing et al., 2003). The second type of interneuron (B4/5) fires after CBI-3 activity ceases (i.e., during the radula retraction phase of the motor program). Effects of CBI-3 on B4/5 are APGWamide mediated and are persistent in that they are manifested for several seconds after neural activity ceases. Our data suggest, however, that this inhibitory effect is not as persistent as excitatory effects induced by FCAP/CP-2 release (e.g., they do not last for minutes). Possibly this reflects the fact that the effects of FCAP/CP-2 and APGWamide are mediated by different second messenger systems.

The difference in the dynamics makes it possible for ingestive activity to be induced either *via* the creation of a relatively persistent state, or relatively dynamically *via* a mechanism that would permit a quick return to an intermediate network configuration. It is possible that *Aplysia* utilizes both types of feeding under physiological conditions. For example, the persistent ingestive state would presumably be beneficial when animals encounter patches of seaweed that can be consumed without any type of switching behavior. In contrast, switching behavior is likely to be most important when there are problems with food intake. For example, in a laboratory setting, switching is observed when animals are given strips of seaweed that are attached to a substrate (Proekt et al., 2008). In this situation, feeding responses can be mixed. Ingestive responses can be followed by egestive responses so that the food that cannot be ingested is removed from the buccal cavity. After inedible food is egested there is a return to ingestion. The animals that we used in this study were adults that had developed in their natural environment. Possibly the fact that ingestive priming was observed in some preparations and not others is a reflection of differences in previous history. Preparations in which we observed ingestive priming might have come from animals that had most recently experienced an environment in which feeding was possible without switching behavior. In contrast, preparations in which priming did not occur may have come from an environment where frequent switching was necessary.

In summary, in a number of systems network activity is triggered by the coactivation of multiple projection neurons. In some cases, this is a reflection of a modular circuit design. In this study, we identify an alternative possibility. We demonstrate that it can determine how rapidly motor activity can be reconfigured.

## Data Availability Statement

The raw data supporting the conclusions of this article will be made available by the authors, without undue reservation.

## Author Contributions

CGE, MAB, MHP, KRW, and ECC contributed to the conception and design of the study. CGE and ECC performed the statistical analysis. ECC wrote the first draft of the manuscript. Experiments were performed by CGE. All authors contributed to the article and approved the submitted version.

## Conflict of Interest

The authors declare that the research was conducted in the absence of any commercial or financial relationships that could be construed as a potential conflict of interest.
